# Terminalin from African Mango (*Irvingia gabonensis*) Stimulates Glucose Uptake through Inhibition of Protein Tyrosine Phosphatases

**DOI:** 10.3390/biom12020321

**Published:** 2022-02-17

**Authors:** Sun-Young Yoon, Jinsoo Kim, Bum Soo Lee, Su Cheol Baek, Sang J. Chung, Ki Hyun Kim

**Affiliations:** 1 Department of Cosmetic Science, Kwangju Women’s University, Gwangju 62396, Korea; dalae1104@gmail.com; 2 School of Pharmacy, Sungkyunkwan University, Suwon 16419, Korea; neto543@naver.com (J.K.); kosboybs@naver.com (B.S.L.); schii513@naver.com (S.C.B.); 3 Department of Biopharmaceutical Convergence, Sungkyunkwan University, Suwon 16419, Korea

**Keywords:** protein tyrosine phosphatases (PTPs), PTPN1, PTPN9, PTPN11, PTPRS, type 2 diabetes, terminalin, *Irvingia gabonensis*, catalytic activity, glucose uptake

## Abstract

Protein tyrosine phosphatases (PTPs), along with protein tyrosine kinases, control signaling pathways involved in cell growth, metabolism, differentiation, proliferation, and survival. Several PTPs, such as PTPN1, PTPN2, PTPN9, PTPN11, PTPRS, and DUSP9, disrupt insulin signaling and trigger type 2 diabetes, indicating that PTPs are promising drug targets for the treatment or prevention of type 2 diabetes. As part of an ongoing study on the discovery of pharmacologically active bioactive natural products, we conducted a phytochemical investigation of African mango (*Irvingia gabonensis*) using liquid chromatography–mass spectrometry (LC/MS)-based analysis, which led to the isolation of terminalin as a major component from the extract of the seeds of *I. gabonensis*. The structure of terminalin was characterized by spectroscopic methods, including one-dimensional (1D) and two-dimensional (2D) nuclear magnetic resonance (NMR) and high-resolution (HR) electrospray ionization (ESI) mass spectroscopy. Moreover, terminalin was evaluated for its antidiabetic property; terminalin inhibited the catalytic activity of PTPN1, PTPN9, PTPN11, and PTPRS in vitro and led to a significant increase in glucose uptake in differentiated C2C12 muscle cells, indicating that terminalin exhibits antidiabetic effect through the PTP inhibitory mechanism. These findings suggest that terminalin derived from African mango could be used as a functional food ingredient or pharmaceutical supplement for the prevention of type 2 diabetes.

## 1. Introduction

Protein tyrosine phosphorylation plays an important role in signal transduction processes involved in cell growth, metabolism, differentiation, and survival [1]. Protein tyrosine phosphatases (PTPs) along with protein tyrosine kinases control the phosphorylation of tyrosine residues [2]. PTPs serve as important regulators of signaling events and regulate tyrosine phosphorylation by catalyzing the removal of phosphate groups from phosphorylated tyrosine residues in proteins [3,4]. PTPs are involved in human diseases such as obesity, diabetes, cancer, and inflammatory diseases [5,6]. Diverse PTPs, such as PTPN1, PTPN2, PTPN9, PTPN11, PTPRS, and DUSP9, have been reported to attenuate insulin signaling and trigger type 2 diabetes, indicating that PTPs are promising drug targets for the treatment or prevention of type 2 diabetes [6,7,8].

*Irvingia gabonensis* (Aubry-Lecomte ex O’Rorke) Baill., also known as “African mango” or “bush mango”, belonging to Irvingiaceae, is a herbaceous and multipurpose fruit tree native to tropical Africa. *I. gabonensis* has been used as a source of timber and to make utensils, and its fruits have been mostly used as food and traditional medicine [9,10,11]. *I. gabonensis* fruit is similar to mango, and called African mango or “wild mango”; recently, the fruit has been reported to have a remarkable effect on weight management [12], making it a popular ingredient in the health food industry. The kernels or seeds of *I. gabonensis* fruit mainly constitute an important part of the diet in West and Central Africa and provide carbohydrates and proteins [12]. The seeds of *I. gabonensis* fruit can be eaten raw or roasted and are also used in food preparations. The seeds are reported to contain high-soluble fibers that can delay gastric emptying and act as a laxative [12]. In previous pharmacological studies, the seeds have shown the potential to improve blood sugar levels in diabetes [13]. Moreover, the aqueous extract of *I. gabonensis* seeds has been reported to cause a dose- and time-dependent decrease in the levels of urea, uric acid, creatine, total cholesterol, alkaline acid, and prostatic acid phosphatases in male guinea pigs in an in vivo study [14]. Treatment with *I. gabonensis* seed extract in obese patients had a positive effect on obesity management, including reducing total cholesterol, triglyceride, low-density lipoprotein (LDL), and blood glucose levels and increasing high-density lipoprotein (HDL)-cholesterol level [15]. Phytochemical analysis of *I. gabonensis* seed extract has identified the presence of diverse constituents, including steroids, flavonoids, alkaloids, cardiac glycosides, volatile oils, terpenoids, tannins, and saponins [16]; moreover, *I. gabonensis* seeds have been reported to contain a significant amount of gallotannins that have high antioxidant capacity [17]. Thus, the presence of diverse bioactive compounds in *I. gabonensis* seeds may lead to the discovery of new bioactive phytochemicals. 

As part of an ongoing research work to discover bioactive natural products from diverse natural resources [18,19,20,21,22,23], we explored phytochemicals from an aqueous extract of *I. gabonensis* seeds. The phytochemical analysis of the extract aided by liquid chromatography–mass spectrometry (LC/MS)-based analysis led to the isolation of one main component, terminalin. The structure of terminalin was identified by nuclear magnetic resonance (NMR) spectroscopy and high-resolution electrospray ionization mass spectrometry (HR-ESIMS), and to the best of our knowledge, the complete NMR assignment of terminalin was reported for the first time in this study. In this study, we report the isolation and chemical identification of terminalin and the experiments performed to evaluate its antidiabetic property. Our cell-based studies indicated that terminalin could be a novel potential therapeutic candidate for the treatment of type 2 diabetes.

## 2. Materials and Methods 

### 2.1. General Experimental Procedures

UV spectra were acquired using Agilent 8453 UV–visible spectrophotometer (Agilent Technologies, Santa Clara, CA, USA). NMR spectra were acquired using Varian UNITY INOVA 800 NMR spectrometer (Varian, Palo Alto, CA, USA) operating at 800 MHz (^1^H) with chemical shifts reported in ppm (δ). Preparative high-performance liquid chromatography (HPLC) was performed using Waters 1525 Binary HPLC pump equipped with Waters 996 Photodiode Array Detector (Waters Corporation, Milford, CT, USA). Diaion HP-20 (Mitsubishi Chemical, Tokyo, Japan) was used for open-column chromatography. Semi-preparative HPLC was performed using Shimadzu Prominence HPLC System equipped with SPD-20A/20AV Series Prominence HPLC UV–Vis Detector (Shimadzu, Tokyo, Japan). LC/MS analyses were performed using Agilent 1200 Series HPLC System (Agilent Technologies, Santa Clara, CA, USA) equipped with a diode array detector and 6130 Series ESI mass spectrometer with an analytical Kinetex HPLC column (4.6 × 100 mm, 3.5 μm). Merck pre-coated silica gel F254 plates and RP-18 F254s plates were used for thin-layer chromatography (TLC). Spots were detected on TLC plates under UV light or by heating after spraying with anisaldehyde sulfuric acid.

### 2.2. Plant Material

The seeds of *I. gabonensis* were provided by the Korean health functional food company FromBIO Co., Ltd, Suwon, Korea. in June 2019. The material was authenticated by one of the authors (K. H. K.). A voucher specimen of the material (WM-FB-2019-06) was deposited at the R&D Center, FromBIO Co., Ltd.

### 2.3. Extraction and Isolation

The seeds (500 g) of *I. gabonensis* were dried, crushed, and then extracted twice with twofold volume of distilled water at 30 °C for 24 h. The extract was filtered using a centrifuge, and the filtrate was concentrated in vacuo using a rotary evaporator at 25–30 °C. The resultant extract was freeze dried to obtain the crude extract powder (35 g). With reference to an in-house-built UV library, LC/MS analysis of the crude extract indicated the presence of an unknown main peak with *m*/*z* 601.0 [M-H]^−^ and one major component, ellagic acid with *m*/*z* 301.0 [M-H]^−^ in the crude extract (Figure S7). To identify the unknown peak, the crude extract (5.0 g) was subjected to Diaion HP-20 column in 100% H_2_O to eliminate the sugar portion, and fraction M was obtained by elution with 100% MeOH. The fraction M (2.5 g) was separated by preparative reversed-phase HPLC using a gradient solvent system of MeOH-H_2_O (10–100% MeOH in 50 min, flow rate of 5 mL/min) to obtain four fractions (M1–M4). Subsequent LC/MS analysis of the fractions derived from HPLC separation indicated the presence of an unknown peak with *m*/*z* 601.0 [M-H]^−^ in the fraction M3. Finally, the fraction M3 (90 mg) was purified by semi-preparative reversed-phase HPLC with 40% MeOH/H_2_O (flow rate of 2 mL/min) to yield terminalin (*t*_R_ = 18.5 min, 50 mg).

#### Terminalin

Bright-yellow powder; UV (MeOH) λ_max_ (log ε) 382 (1.5), 258 (4.3) nm; IR (KBr) ν_max_ 3350, 2942, 1715, 1583, 1502, and 1025 cm^−1^; ^1^H and ^13^C NMR (800 and 200 MHz, respectively) (Table 1); negative ESIMS *m*/*z* 601.1 [M-H]^−^; positive HR-ESIMS *m*/*z* 603.0045 [M+H]^+^ (calculated for C_28_H_11_O_16_, 603.0047).

### 2.4. Measurement of the Effect of Terminalin on PTP Catalytic Activity 

Activity assays of purified PTPs using 6,8-difluoro-4-methylumbelliferyl phosphate (DiFMUP), a widely used PTP substrate, were performed as previously described [24,25]. To obtain *K*_M_ values, PTPN1 (0.5 nM), PTPN9 (0.1 nM), PTPN11 (1.5 nM), PTPRS (0.45 nM), and PTPRF (0.6 nM) were added to the reaction buffer (150 mM NaCl, 2.5 mM dithiothreitol, 0.01% Triton X-100, and 20 mM Tris; pH 6.0 for PTPN1, PTPN11, PTPRS, and PTPRF or pH 7.0 for PTPN9) containing DiFMUP in black 96-well microplates. Fluorescence intensity was measured continuously for 10 min (excitation/emission wavelengths = 355/460 nm) using VictorTM X4 multilabel plate reader (Perkin Elmer, Norwalk, CT, USA), and *K*_M_ values were determined using Lineweaver–Burk plots. To estimate the inhibitory effect of terminalin on PTPs, PTPs were added to solutions containing 20 μM terminalin in reaction buffer with DiFMUP (2 × *K*_M_).

### 2.5. Cell Culture

The method used for culturing C2C12 muscle cells obtained from the American Type Culture Collection (ATCC; Manassas, VA, USA) has been described previously [26]. C2C12 muscle cells were cultured in high-glucose Dulbecco’s modified Eagle’s medium (DMEM; Welgene, Gyeongsan-si, South Korea) supplemented with 15% fetal bovine serum (FBS; Welgene) and antibiotic–antimycotic solution (Welgene).

### 2.6. Cell Differentiation

The method used for differentiating C2C12 muscle cells has been described previously [26]. When C2C12 muscle cells reached 70% confluency, they were cultured in DMEM supplemented with 2% horse serum (Thermo Fisher Scientific Korea Ltd., Seoul, Korea) and antibiotic–antimycotic solution (Welgene) for six days. In all experiments, the culture medium was changed every alternate day.

### 2.7. Cell Viability Assay

To measure cell viability, EX-Cytox (DOGEN Bio., Seoul, Korea) was used according to the manufacturer’s instructions. Differentiated C2C12 muscle cells were treated with various concentrations of terminalin for 48 h, and cell viability was measured at 450 nm using a microplate reader (VictorTM X4, PerkinElmer, Waltham, MA, USA).

### 2.8. Glucose Uptake Assay

The methods used for measuring glucose uptake in differentiated C2C12 muscle cells have been described previously [25]. Differentiated C2C12 muscle cells were cultured in low-glucose DMEM (Gibco BRL, More) for 16 h and then incubated with 40 µM terminalin, control (0.1% dimethyl sulfoxide), or 0.1 µM insulin (positive control) in glucose-depleted DMEM (Gibco BRL) for 6 h (terminalin and control) or 30 min (insulin). Next, the cells were treated with a fluorescent glucose indicator, 100 µM 2-[N-(7-nitrobenz-2-oxa-1,3-diazol-4-yl)amino]-2-deoxyglucose (2-NBDG; Thermo Fisher Scientific), for 30 min. After washing with phosphate-buffered saline (PBS; Welgene), fluorescence intensity of the cells at excitation/emission (485/535 nm) was measured using a fluorescence microplate reader (VictorTM X4).

### 2.9. Statistical Analysis

Statistical significance (*p* < 0.05) was determined using two-tailed unpaired *t*-test (GraphPad Software, San Diego, CA, USA). All experiments were conducted independently thrice. 

## 3. Results and Discussion

### 3.1. Isolation of Terminalin 

**Figure 1:**
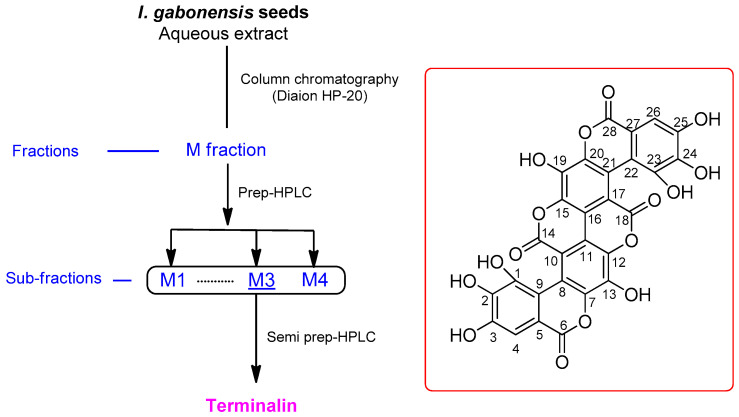
Schematic representation of the isolation of terminalin and its chemical structure.

*I. gabonensis* seeds were dried, crushed, and then extracted with water at 30 °C to obtain the crude aqueous extract by rotary evaporation and freeze-drying. LC/MS-based analysis of the crude extract was carried out by reference to an in-house-built UV library database, which revealed the presence of an unknown main peak with *m*/*z* 601.0 [M-H]^−^ and a unique UV spectrum of the extended conjugated system (λ_max_ 382 and 258 nm), and one major component, ellagic acid in the crude extract (Figure S7), where ellagic acid was confirmed without isolation by reference to our in-house UV library and the molecular ion with *m*/*z* 301.0 [M-H]^−^ detected by LC/MS. LC/MS/UV-guided fractionation of the crude extract and semi-preparative HPLC resulted in the isolation of the unknown main peak with *m*/*z* 601.0 [M-H]^−^ (Figure 1). Structural characterization of the isolated compound was performed by NMR and MS analyses, and the compound was identified as terminalin (Figure 1). 

### 3.2. Structural Elucidation of the Isolated Compound 

The isolated compound was a bright-yellow powder. Its molecular formula was determined as C_28_H_10_O_16_, based on the HR-ESIMS data, which showed the molecular ion peak with *m*/*z* 603.0045 [M+H]^+^ (calculated for C_28_H_11_O_16_, 603.0047) in the positive mode. The IR spectrum displayed distinctive absorption bands for the hydroxy group (3350 cm^−1^), ester carbonyl functional unit (1715 cm^−1^), and aromatic rings (1583 and 1502 cm^−1^), and the UV spectrum showed a unique UV absorption (λ_max_ 382 and 258 nm), implying an extended conjugated system in the molecule. The ^1^H NMR data (Table 1) of the compound comprised only one sharp singlet at *δ*_H_ 7.52 in DMSO-*d_6_*. The ^13^C NMR spectrum (Table 1) showed fourteen carbon resonances (*δ*_C_ 159.5–107.2) rather than twenty-eight carbon signals established by the HR-ESIMS data, which suggested that the compound has a symmetric structure or dimeric structure. The ^13^C NMR chemical shifts comprised three sets of signals, including the first group for two carbonyl carbons at *δ*_C_ 159.5 and 158.3, the second group for six oxygen-bearing carbons at *δ*_C_ 148.3–136.3, and the third group for the remaining six-carbon signals corresponding to the remaining quaternary carbon and the proton-bearing carbons at *δ*_C_ 123.3–107.2. Comprehensive analysis of the NMR spectral data suggested that the compound could be a dimeric analog of ellagic acid. Based on the NMR and physical data described in previous studies [27,28], where the reported NMR data of terminalin showed a good correlation with those obtained from the isolated compound; the isolated compound was identified unambiguously as terminalin (Figure 1). 

**Figure 2:**
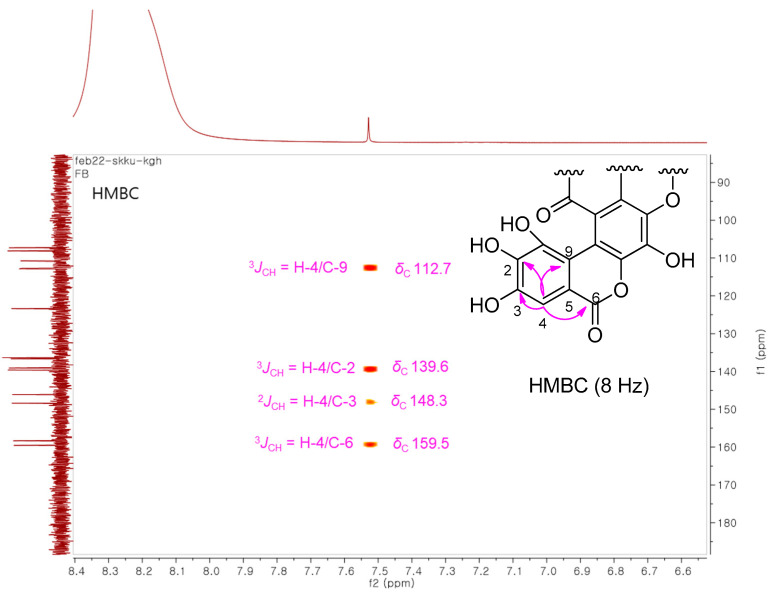

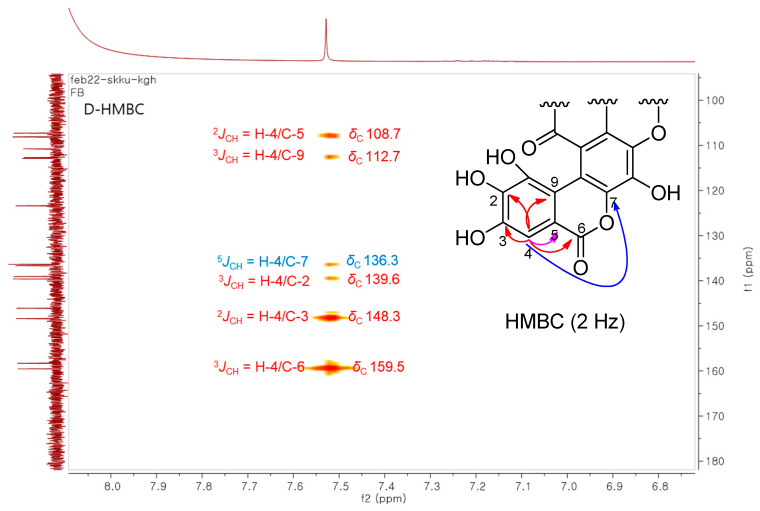
The key HMBC correlations for terminalin.

Although 2D NMR experiment for ^13^C−^1^H interactions was redundant for determining the structure of terminalin because only one ^1^H proton signal was detected and terminalin has a proton-deficient structural scaffold, we conducted an additional heteronuclear multiple-bond correlation (HMBC) experiment to complete the NMR assignment of terminalin (Table 1). To the best of our knowledge, the NMR data of terminalin have been reported [27,28]; however, the NMR signals have not yet been individually assigned. Considering the structure of terminalin that presents a proton-deficient structural scaffold, it would be difficult to define the structure using only a standard HMBC experiment (optimized for ^n^*J*_CH_ ≃ 8 Hz) that generally provides two- and three-bond heteronuclear correlations. The decoupled HMBC (D-HMBC) experiment with an elongated long-range delay optimized for ^n^*J*_CH_ = 2 Hz allows long-range ^4^*J* and occasionally ^5^*J* and ^6^*J* couplings to be visible [29]. In this study, we employed D-HMBC experiment as well as standard HMBC, which allowed us to distinguish the four- and five-bond ^1^H−^13^C correlations from the two- and three-bond correlations (Figure 2). These HMBC data showed the key correlations of H-4 with C-3 (^2^*J*), C-5 (^2^*J*), C-2 (^3^*J*), C-6 (^3^*J*), and C-9 (^3^*J*), and C-7 (^5^*J*), enabling the NMR assignment of terminalin. The remaining carbon signal assignment was completed based on the structure-based optimal estimation by Predict ^13^C NMR Shifts function in ChemDraw 12.0. The present study reports the complete NMR assignment of terminalin for the first time.

### 3.3. Terminalin Shows Inhibitory Effect on PTPs Relevant to Insulin Resistance

Terminalin, also known as gallagyldilactone, is a polyphenol compound mainly found in pomegranate. Pomegranate juice, which contains terminalin, ellagic acid, and gallic acid, exhibits beneficial health properties such as antioxidant, anti-inflammatory, and anticancer activities [30,31,32,33]. However, the antidiabetic effect of terminalin and its targets has not been investigated.

**Figure 3:**
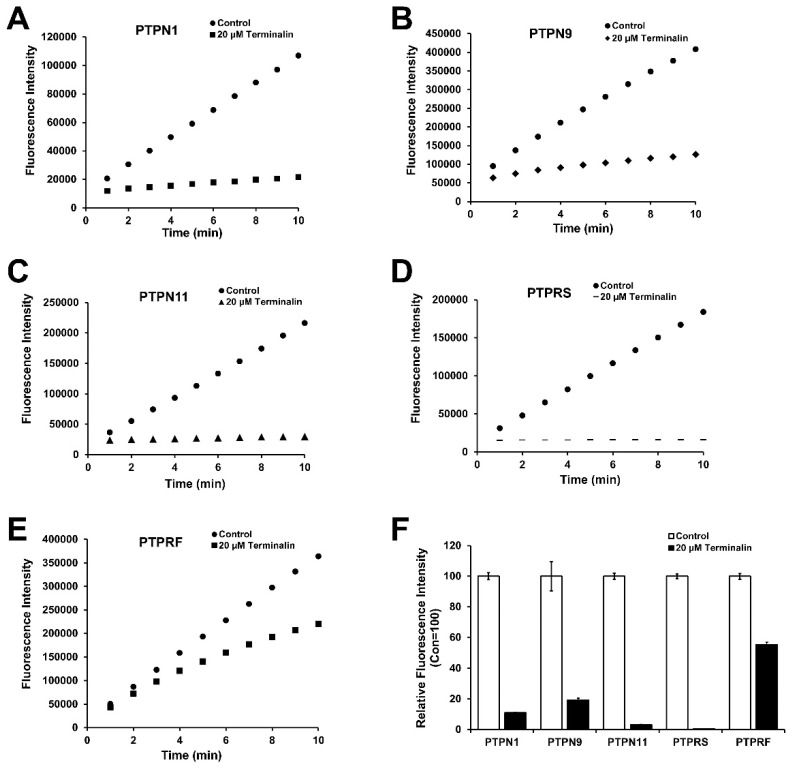
Terminalin inhibits the catalytic activity of PTPs. (**A**–**E**) The catalytic activities of PTPs against terminalin were measured using DiFMUP, a widely used PTP substrate. Progress curves showing catalytic activity of PTPs by 20 µM terminalin and control. (**F**) Relative fluorescence intensity of PTPs against terminalin and control.

PTPs along with protein tyrosine kinases control signaling pathways involved in cell growth, metabolism, differentiation, proliferation, and survival [1,34]. It has been shown that several PTPs, such as PTPN1, PTPN2, PTPN6, and PTPN9, modulate the insulin signaling pathway by dephosphorylating the insulin receptor [6,35]. Some PTPs, including PTPN1, PTPN2, PTPN9, PTPN11, PTPRS, and DUSP9, are associated with type 2 diabetes; therefore, recognition of agents that can inhibit PTPs may be a strategy to identify promising drugs with antidiabetic properties. Many natural products have been identified to treat or prevent various human diseases via inhibition of PTPs, for instance, illudalic acid inhibited the catalytic activity of human leukocyte common antigen-related PTP involved in the pathogenesis of insulin resistance [6,36]. To identify the targets of terminalin, PTPN1, PTPN9, PTPN11, PTPRF, and PTPRS were overexpressed in *Escherichia coli* and purified using a cobalt-affinity resin. The catalytic activities of PTPs against terminalin were evaluated using DiFMUP, a widely used PTP substrate. The kinetic constants were determined to measure the enzymatic activities of these PTPs (Table 2). Terminalin showed more than 80% inhibition of PTPN1, PTPN9, PTPN11, and PTPRS, which are relevant to insulin resistance (Figure 3), indicating terminalin as a potential therapeutic candidate for the prevention of type 2 diabetes.

### 3.4. Terminalin Increases Glucose Uptake in C2C12 Cells 

**Figure 4:**
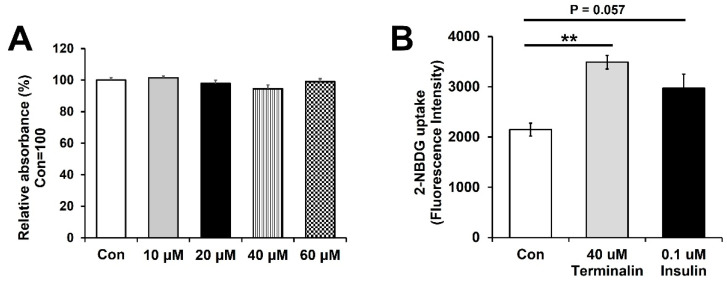
Terminalin increased glucose uptake in differentiated C2C12 muscle cells: (**A**) Differentiated C2C12 muscle cells were treated with the indicated concentrations of terminalin for 48 h, and cell viability was determined using EZ-Cytox assay kit. (**B**) Differentiated C2C12 muscle cells were incubated with 40 µM terminalin, 0.1 µM insulin (positive control), or control (0.1% dimethyl sulfoxide) for 6 h (terminalin and control) or 30 min (insulin). Cells were then treated with 2-NBDG, a fluorescent glucose probe, for 30 min, and the fluorescence intensity of the cells was measured. Results are expressed as the mean ± standard error of the mean (SEM). All experiments were conducted independently three times. Data were analyzed using two-tailed unpaired *t*-test. ** *p* < 0.01 compared to the control group.

To evaluate the antidiabetic property of terminalin, we investigated its effect on glucose uptake in C2C12 muscle cells. Many natural products exhibit potent antidiabetic properties by activating insulin signaling; the herbal medicine calycosin has been shown to significantly improve symptoms of gestational diabetes by rebalancing the insulin sensitivity and inflammatory response [6,37]. In this study, to optimize the concentration of terminalin for the treatment of C2C12 muscle cells, various concentrations of terminalin were applied to the cells for 48 h, and cell viability was measured using EZ-Cytox assay kit. We observed no cytotoxicity in cells treated with 10, 20, or 40 µM terminalin, indicating that these concentrations were suitable for the treatment of the cells (Figure 4A). Next, we evaluated the effect of terminalin on glucose uptake in differentiated C2C12 muscle cells using 2-NBDG, a fluorescent glucose probe. Cells were treated with 40 µM terminalin or 0.1 µM insulin (positive control) for 6 h or 30 min, respectively, and then incubated with 2-NBDG for 30 min. After washing with PBS, fluorescence intensity (excitation/emission = 485/535 nm) of the cells was assessed. Since insulin promotes glucose uptake in muscles and adipose tissue, it was used as a positive control [38]. We observed that incubation with 40 µM terminalin significantly enhanced the fluorescence intensity compared to the control, indicating that terminalin stimulated glucose uptake in differentiated C2C12 muscle cells (Figure 4B). In addition, 0.1 µM insulin treatment induced glucose uptake compared to the control, indicating that the fluorescent glucose probe worked well in our system (Figure 4B). Next, we investigated whether terminalin can enhance AMPK phosphorylation of threonine 172 in differentiated C2C12 muscle cells. Adenosine monophosphate-activated protein kinase (AMPK; a cellular energy sensor) is involved in maintaining glucose homeostasis and the activation of AMPK has been shown to stimulate glucose uptake by promoting the translocation of glucose transporter 4 [39,40]. We used 5-aminoimidazole-4-carboxamide riboside (AICAR; an AMPK activator) as a positive control. Differentiated C2C12 muscle cells were incubated with 40 µM terminalin, control (0.1% dimethyl sulfoxide) or 1 mM AICAR (positive control) for 6 h and Western blotting was performed. The 40 µM terminalin treatment showed a tendency to increase AMPK phosphorylation compared to the control group (Figure S8). In addition, the 1 mM AICAR treatment enhanced AMPK phosphorylation, indicating that Western blotting worked properly (Figure S8). These results indicate that terminalin stimulated glucose uptake through activation of the AMPK signaling pathway.

## 4. Conclusions

PTPs regulate tyrosine phosphorylation associated with signaling events and have been implicated in human diseases such as obesity, diabetes, cancer, and inflammatory diseases [35]. Some PTPs, such as PTPN1, PTPN2, PTPN9, PTPN11, PTPRS, and DUSP9, have been shown to attenuate insulin signaling and trigger type 2 diabetes, indicating that PTPs are promising drug targets for the treatment or prevention of type 2 diabetes [5,6]. In this study, we performed the isolation and chemical identification of terminalin and demonstrated for the first time, to the best of our knowledge, that terminalin inhibited the catalytic activity of PTPN1, PTPN9, PTPN11, and PTPRS, which are relevant to insulin resistance. In addition, terminalin significantly stimulated glucose uptake in C2C12 muscle cells. Taken together, our results suggest that terminalin exhibits an antidiabetic effect through PTP inhibitory mechanism, and it can be used as a functional food ingredient or pharmaceutical supplement for the prevention of type 2 diabetes.

## Figures and Tables

**Table 1:** ^1^H (800 MHz) and ^13^C NMR (200 MHz) data of terminalin in DMSO-*d_6_* (δ ppm) ^a^.

Position	Terminalin	
	*δ*_H_ (*J* in Hz)	*δ*_C_
1, 23		139.0 s
2, 24		139.6 s
3, 25		148.3 s
4, 26	7.52 s	110.7 d
5, 27		108.7 s
6, 28		159.5 s
7, 20		136.3 s
8, 21		123.3 s
9, 22		112.7 s
10, 17		107.2 s
11, 16		112.8 s
12, 15		136.6 s
13, 19		146.1 s
14, 18		158.3 s

^a^*J* values are in Hz and shown in parentheses; ^13^C NMR assignments are based on HMBC experiments.

**Table 2:** Kinetic constants for DiFMUP hydrolysis by PTPs.

	[E] (nM)	*K*_M_ (µM)
PTPN1	0.5	163.1
PTPN9	0.1	200
PTPN11	1.5	74
PTPRS	0.45	56
PTPRF	0.6	150

## Data Availability

Not applicable.

## Supplementary Materials

The following are available online at https://www.mdpi.com/article/10.3390/biom12020321/s1; Figure S1: HR-ESIMS data of terminalin; Figure S2: ^1^H NMR spectrum of terminalin (DMSO-*d_6_*, 800 MHz); Figure S3: ^13^C NMR spectrum of terminalin (DMSO-*d_6_*, 200 MHz); Figure S4: HMBC spectrum of terminalin (DMSO-*d_6_*); Figure S5: D-HMBC spectrum of terminalin (DMSO-*d_6_*); Figure S6: UV spectrum of terminalin; Figure S7: LC/MS chromatogram of the crude extract (detected at 230 nm); Figure S8: Effects of terminalin on C2C12 muscle cells; Table S1: LC-MS conditions for the crude extract.
