# Supplementary material for: Terminalin from African Mango (Irvingia gabonensis) Stimulates Glucose Uptake through Inhibition of Protein Tyrosine Phosphatases
Source: Biomolecules. 2022 Feb 17;12(2):321. doi: 10.3390/biom12020321 (PMC8869479; doi:10.3390/biom12020321)
Supplement: Supplementary file 1 [file biomolecules-12-00321-s001.zip › biomolecules-1587098-supplementary.pdf]

Supplementary data

Terminalin from African Mango (*Irvingia gabonensis*) Stimulates Glucose Uptake through Inhibition of Protein Tyrosine Phosphatases

Sun-Young Yoon [1,†], Jinsoo Kim [2,3,†], Bum Soo Lee [2,†], Su Cheol Baek [2], Sang J. Chung [2,3,*] and Ki Hyun Kim [2,*]

[1] Department of Cosmetic Science, Kwangju Women's University, Gwangju 62396, Korea

[2] School of Pharmacy, Sungkyunkwan University, Suwon 16419, Republic of Korea

[3] Department of Biopharmaceutical Convergence, Sungkyunkwan University, Suwon 16419, Republic of Korea

[†] These authors contributed equally to this work.

[*]Corresponding author. Phone: +82-31-290-7703 (S.J.C). E-mail address: sjchung@skku.edu (S.J.C)

[*]Corresponding author. khkim83@skku.edu (K.H.K.); Tel.: +82-31-290-7341 (C.P.); +82-31-290-7700 (K.H.K.)

Supporting Information Contents:

Materials and Methods: Western blotting ……………..………..……..………………………………………………………….S3
Figure S1. HR-ESIMS data of terminalin ……………..……………………………………………………………….S4
Figure S2. ^{1}H NMR spectrum of terminalin (DMSO-d_6, 800 MHz)………………………………………………………….S5
Figure S3. ^{13}C NMR spectrum of terminalin (DMSO-d_6, 200 MHz)………………….…………………..………………S6
Figure S4. HMBC spectrum of terminalin (DMSO-d_6)………………………………………………………………S7
Figure S5. D-HMBC spectrum of terminalin (DMSO-d_6)……………………………………………………………S8
Figure S6. UV spectrum of terminalin ………………………………………………………………………S9
Figure S7. LC/MS chromatogram of the crude extract (detected at 230 nm)………………………………………………… S10
Figure S8. Effects of terminalin on C2C12 muscle cells…………………………..……………………..………………………… S11
Table S1. LC-MS conditions for the crude extract…………………………………………………………………..S12

Materials and Methods

Western blotting

Proteins were extracted using RIPA buffer (Sigma-Aldrich, Saint Louis, Missouri, USA) containing protease inhibitor cocktail and phosphatase inhibitor cocktail (Sigma-Aldrich). Next, the proteins were separated by electrophoresis on a 10% sodium dodecyl sulfate-polyacrylamide gel and transferred to a polyvinylidene fluoride (PVDF) membrane (Merck KGaA, Darmstadt, Germany) using a wet transfer system. The membranes were incubated overnight at 4°C with primary antibodies. The primary antibodies used were as follows: anti-total AMPK, anti-phosphorylated AMPK, anti-total Akt, anti-phosphorylated Akt (Cell Signaling Technology, Beverly, MA, USA), and anti-β-actin (AbFrontier, Seoul, South Korea). Next, the membranes were incubated with an anti-rabbit-IgG-horseradish peroxidase-conjugated secondary antibody (Santa Cruz Biotechnology). Antibody-antigen complexes were detected using enhanced chemiluminescence reagents (GE Healthcare Korea, Incheon, South Korea). Chemiluminescent images were acquired using a LuminoGraph II Imaging System (ATTO Corporation, Tokyo, Japan).

Figure S1. HR-ESIMS data of terminalin

Compound Identification Results: Cpd 336: C28 H10 O16; 3.502

ID Techniques Applied

MFG

DBE	Mass (MFG)	Score (MFG)	Diff (abs. ppm)	Best	Name	Diff (mDa)	Formula	ID Source	Species	m/z	Score (Manual ID)	Mass	Score	Diff (ppm)	RT
24	601.9969	99.76	0.38	⦿		0.23	C28 H10 O16	MFG	(M+H)+ (M+Na)+	603.0045 624.9867		601.9971	99.76	0.38	3.502
46	602.0004	35.83	4.87	○		-2.93	C46 H2 O3	MFG	(M+Na)+	624.9867		601.9975	35.83	-4.87	3.502

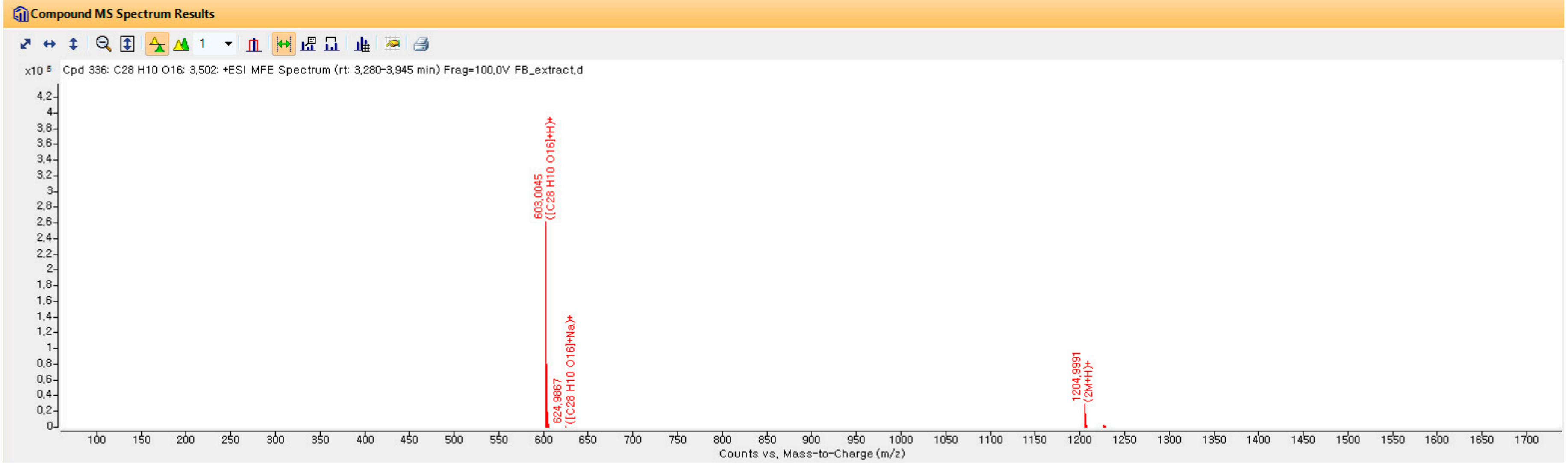

Figure S2. ^{1}H NMR spectrum of terminalin (DMSO-d_6, 800 MHz)

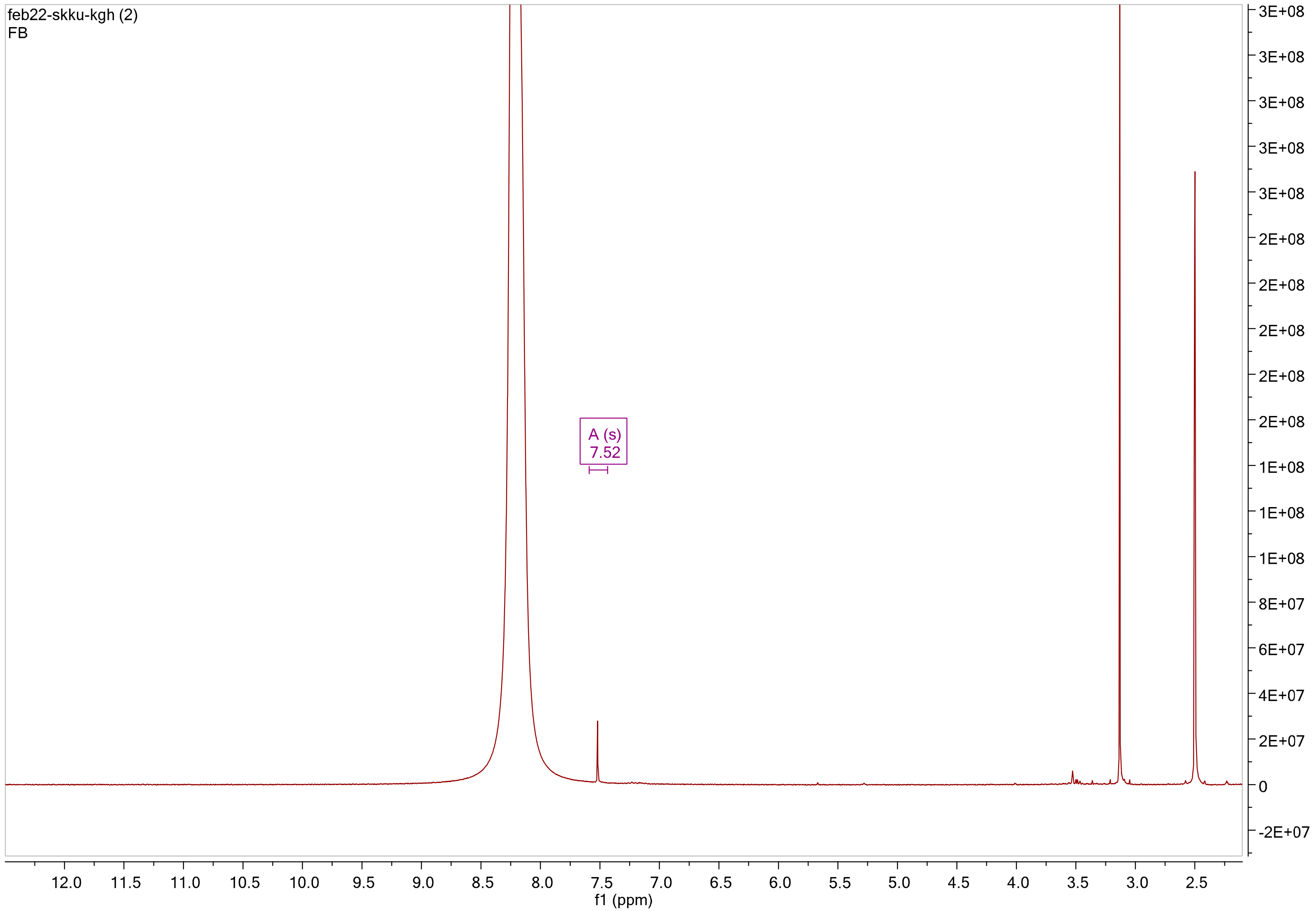

Figure S3. ^{13}C NMR spectrum of terminalin (DMSO-d_6, 200 MHz)

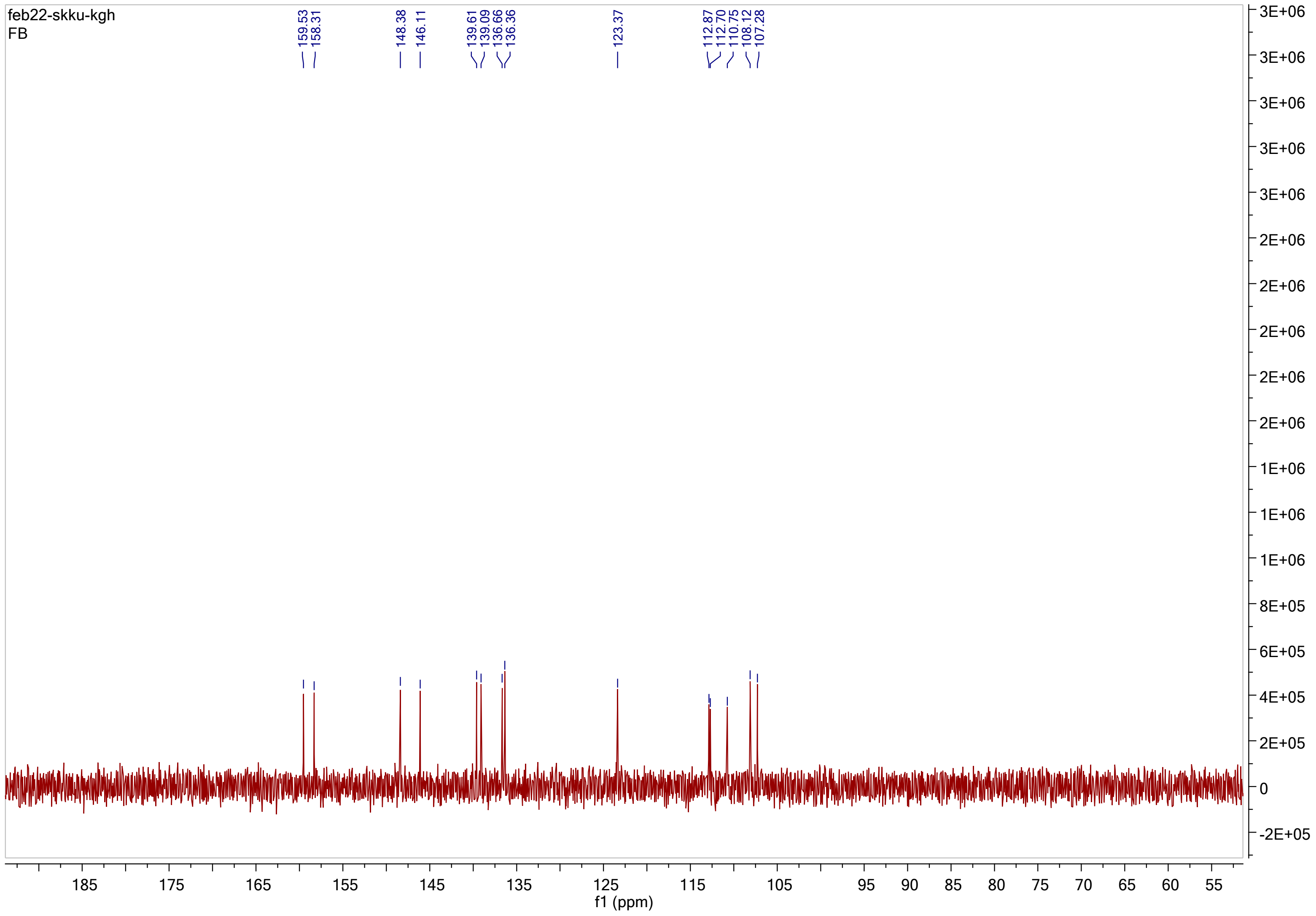

Figure S4. HMBC spectrum of terminalin (DMSO-d_6)

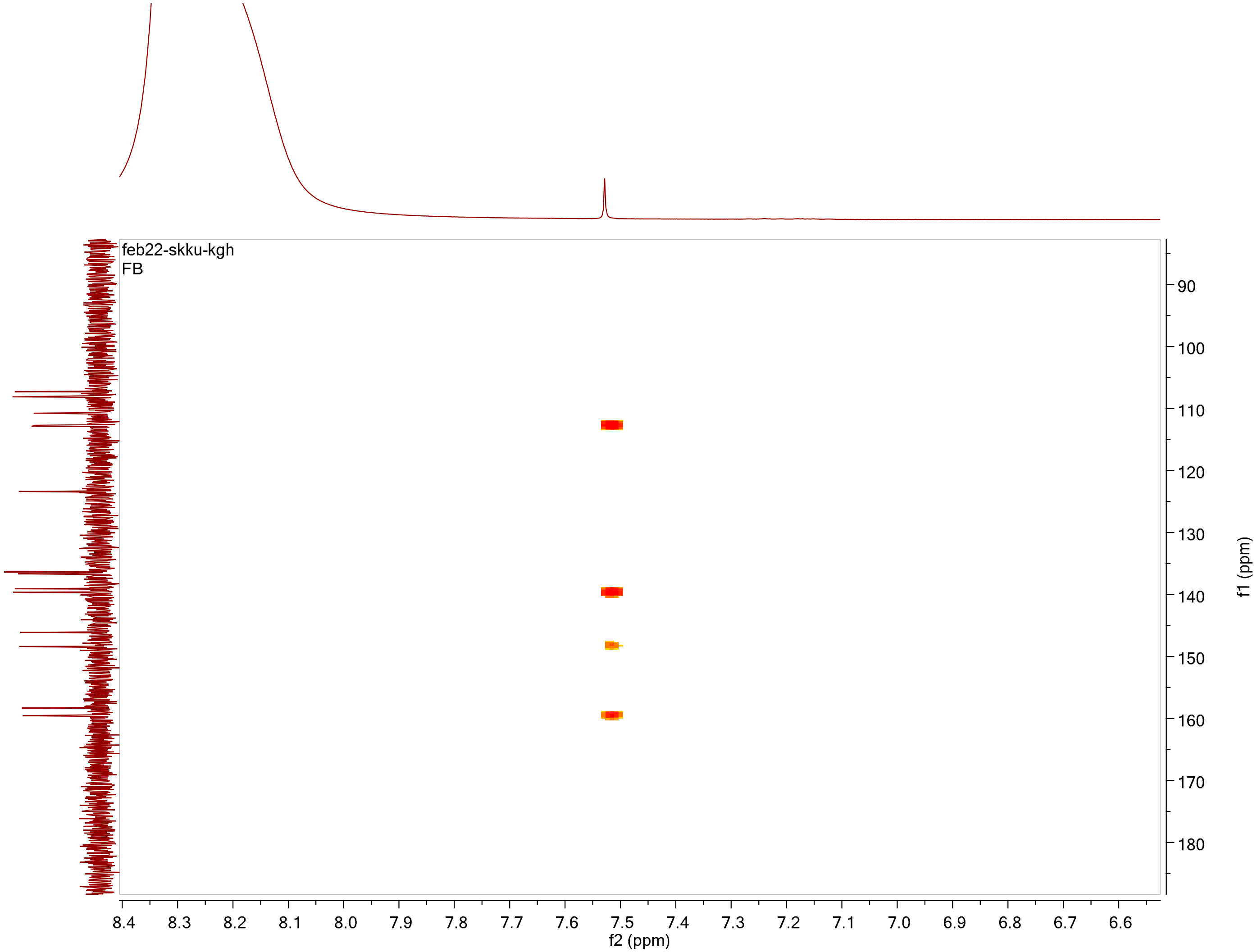

Figure S5. D-HMBC spectrum of terminalin (DMSO-d_6)

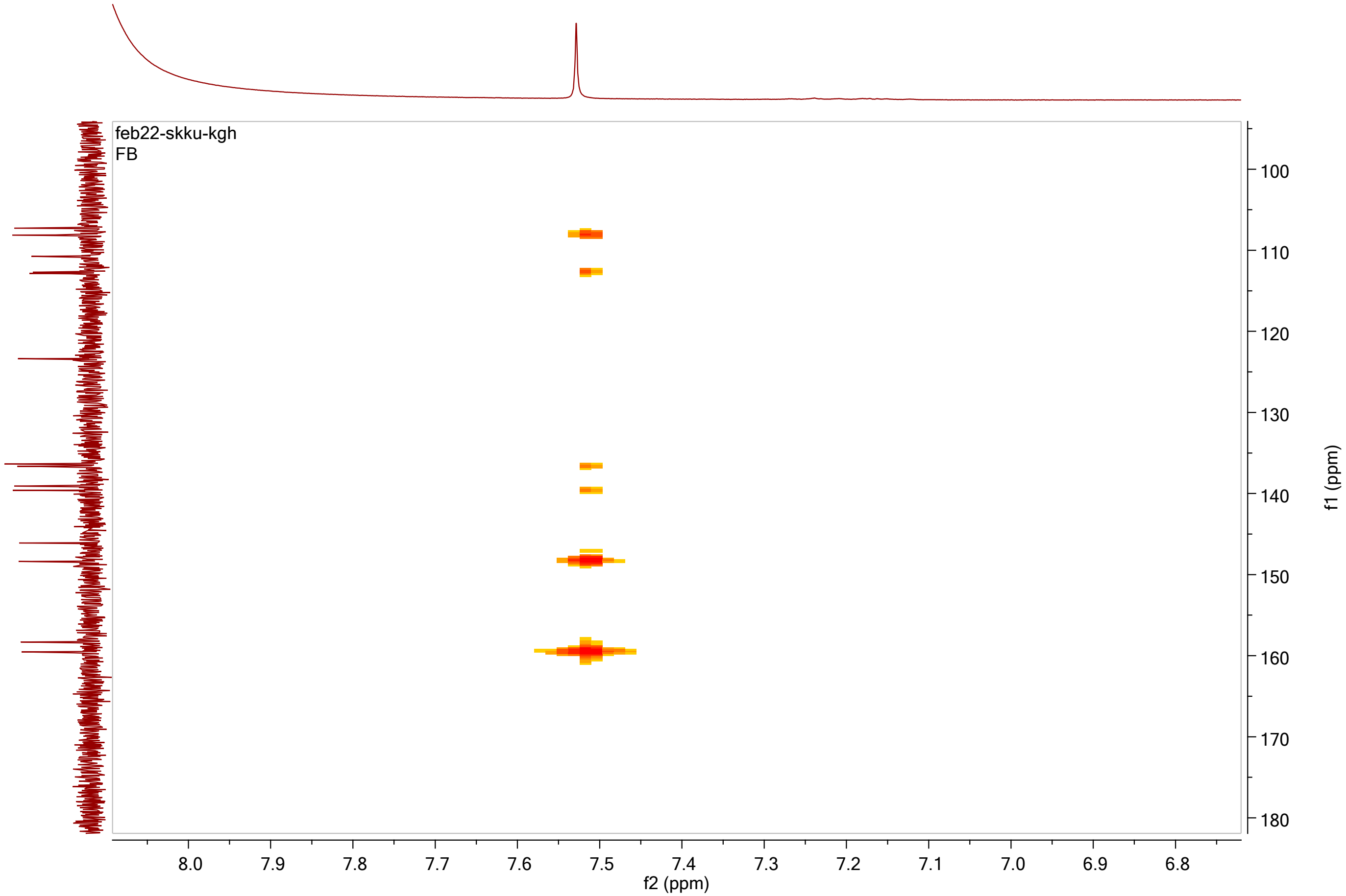

Figure S6. UV spectrum of terminalin

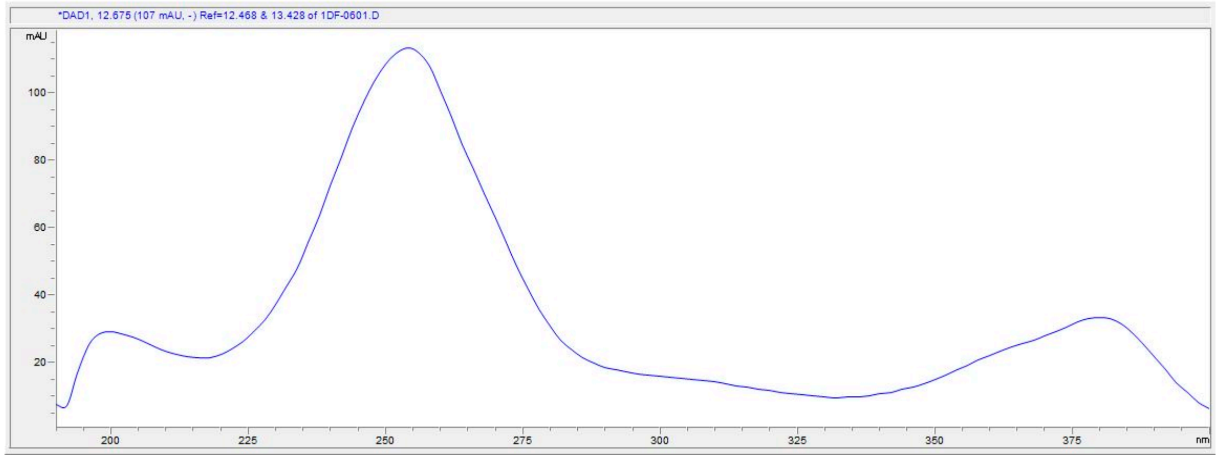

Figure S7. LC/MS chromatogram of the crude extract (detected at 230 nm)

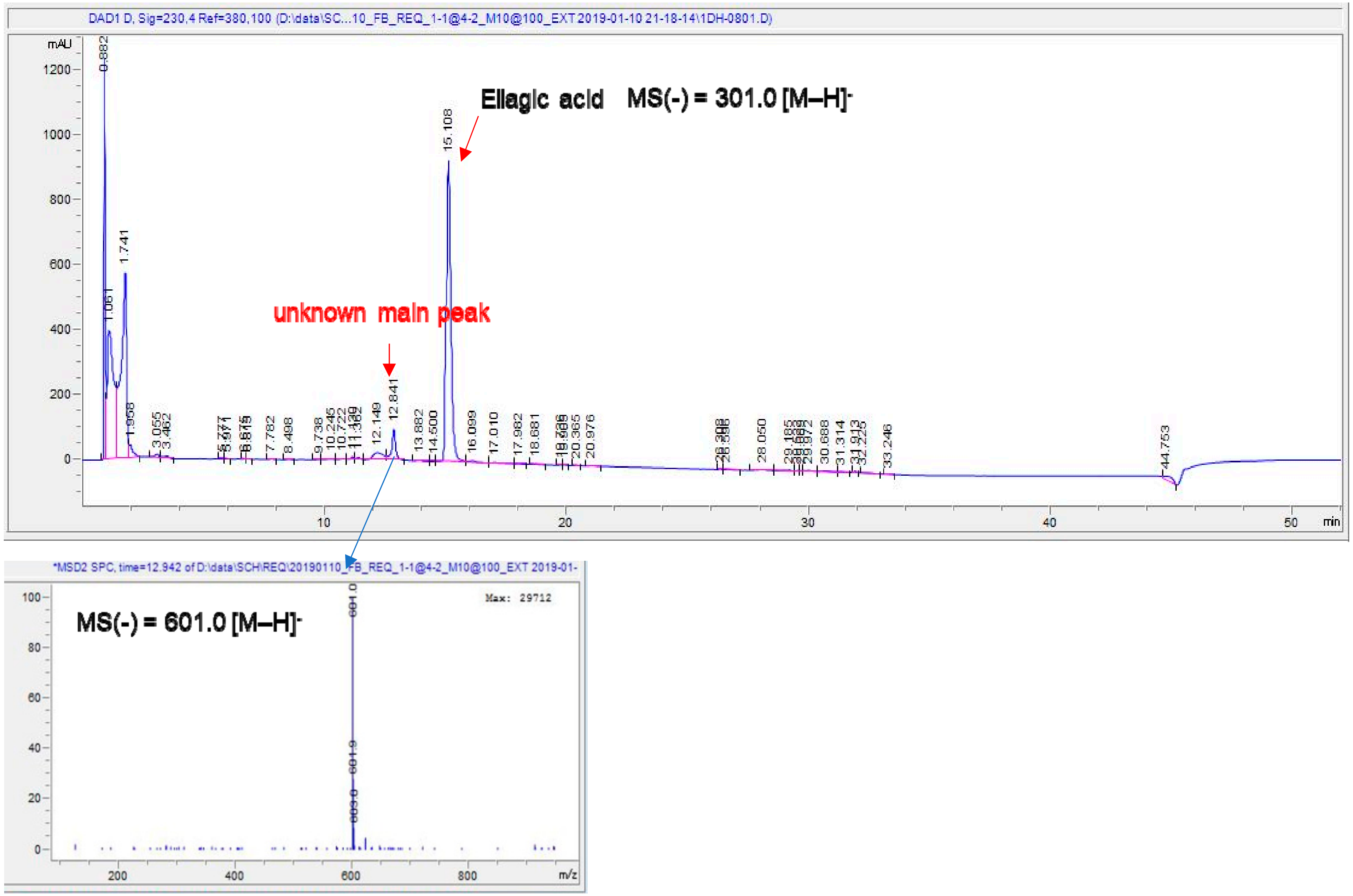

Figure S8. Effects of terminalin on C2C12 muscle cells. Differentiated C2C12 muscle cells were incubated with terminalin, control (0.1% dimethyl sulfoxide), AICAR (positive control; AMPK activator) or insulin (positive control) for 6 h (control, terminalin and AICAR) or 1 h (insulin), and western blotting was performed.

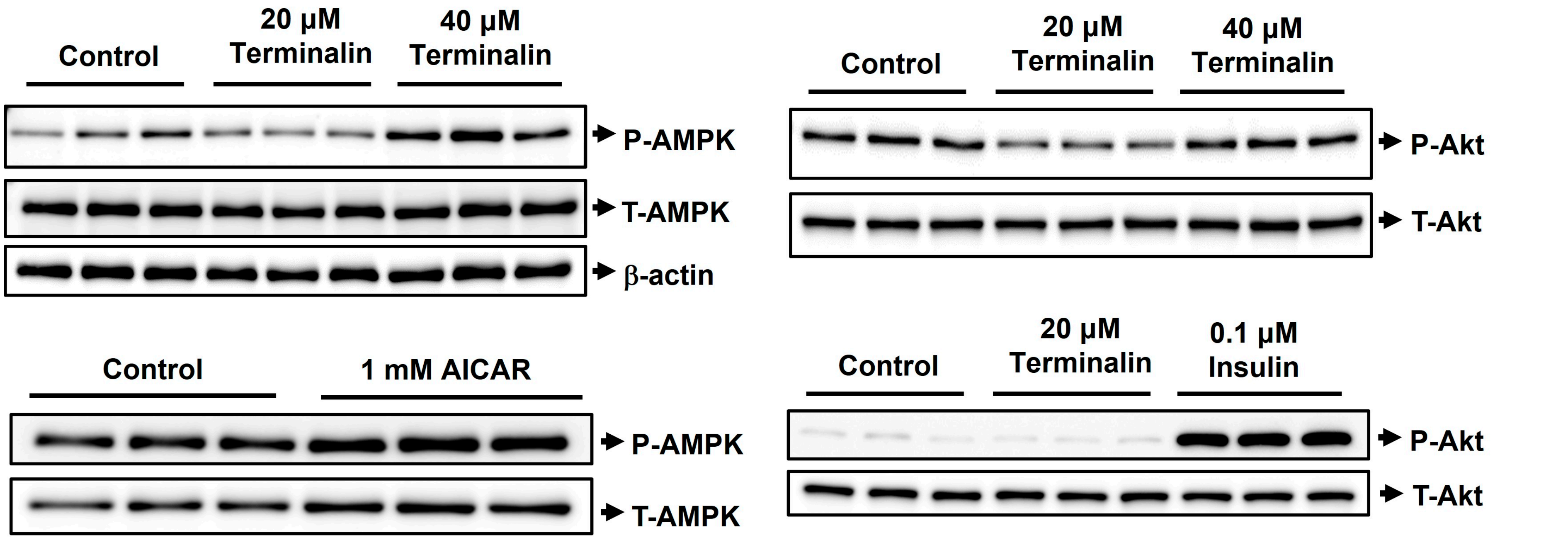

Table S1. LC-MS condition for crude extract

Column	Kinetex C18 column (2.1 × 100 mm, 5 μm; Phenomenex)		
Flow rate	0.3 mL/min		
Injection volume	15 μL		
Column temperature	25°C		
Mobile solvent	A: 0.1% formic acid in water, B: MeOH		
Mobile phase	Time	A (%)	B (%)
	0	90	10
	30	0	100
	31	0	100
	41	0	100
	42	90	10
	52	90	10
